# Esophageal Cancer Patient Network: a scalable platform for integrating patient voices across the research lifecycle

**DOI:** 10.1093/dote/doag046

**Published:** 2026-05-12

**Authors:** Talia Trottenberg, Mariam Hegazy, Sana Astany, Sam Sedighi, Sara Najmeh, Carmen Mueller, Jonathan Spicer, Jonathan Cools-Lartigue, Lorenzo Ferri, R Trafford Crump

**Affiliations:** Division of Thoracic and Upper Gastrointestinal Surgery, Department of Surgery, McGill University, Montreal, QC, Canada; McGill University Health Centre, Montreal, QC, Canada; Division of Thoracic and Upper Gastrointestinal Surgery, Department of Surgery, McGill University, Montreal, QC, Canada; Division of Thoracic and Upper Gastrointestinal Surgery, Department of Surgery, McGill University, Montreal, QC, Canada; Division of Thoracic and Upper Gastrointestinal Surgery, Department of Surgery, McGill University, Montreal, QC, Canada; McGill University Health Centre, Montreal, QC, Canada; Division of Thoracic and Upper Gastrointestinal Surgery, Department of Surgery, McGill University, Montreal, QC, Canada; McGill University Health Centre, Montreal, QC, Canada; Division of Thoracic and Upper Gastrointestinal Surgery, Department of Surgery, McGill University, Montreal, QC, Canada; McGill University Health Centre, Montreal, QC, Canada; Division of Thoracic and Upper Gastrointestinal Surgery, Department of Surgery, McGill University, Montreal, QC, Canada; McGill University Health Centre, Montreal, QC, Canada; Division of Thoracic and Upper Gastrointestinal Surgery, Department of Surgery, McGill University, Montreal, QC, Canada; McGill University Health Centre, Montreal, QC, Canada; Division of Thoracic and Upper Gastrointestinal Surgery, Department of Surgery, McGill University, Montreal, QC, Canada; McGill University Health Centre, Montreal, QC, Canada

**Keywords:** Esophageal Neoplasms, Patient Participation, Community-Based Participatory Research, Research Design, Stakeholder Participation

## Abstract

Esophageal cancer is associated with substantial symptom burden and long-term impacts on quality of life, yet research priorities have historically been shaped with limited direct input from patients and caregivers. Although patient engagement is increasingly recognized as essential for improving the relevance, feasibility, and accessibility of health research, existing national and international frameworks often provide limited guidance on how to operationalize engagement within specific disease contexts. This gap is particularly evidence in esophageal cancer, a relatively low-prevalence disease characterized by complex treatment pathways and heterogeneous patient experiences. To address this need, we established the Esophageal Cancer Patient Network (ECPN), a disease-specific patient engagement initiative designed as a reusable research tool rather than a single-project advisory group. This paper describes the conceptual foundation, governance structure, and implementation strategy of the ECPN. Central to the model is a publicly available Terms of Reference, a continuous engagement framework spanning the full research lifecycle, and clearly defined levels of patient involvement. The ECPN emphasized sustained engagement, enabling patients and caregivers to develop research-related expertise over time through training, mentorship, and repeated collaboration with clinicians and scientists. Designed to support multidisciplinary and international research, the ECPN facilitates collaboration across institutions and healthcare systems while remaining open to participation from patients, caregivers, and researchers beyond a single center. By providing practical infrastructure for sustained, transparent, and disease-specific patient engagement, the ECPN addresses a critical gap between high-level engagement principles and real-world implementation in esophageal cancer research.

## INTRODUCTION

Esophageal cancer is associated with a high symptom burden and significant long-term impacts on patients’ quality of life, including dysphagia, nutritional challenges, fatigue, and psychological distress.^1,2^ Despite advances in surgical and multimodal therapies, many aspects of the patient experience remain insufficiently addressed by research agendas that are largely shaped by clinicians and investigators.^3^ As a result, research priorities often reflect implicit assumptions about patient needs rather than insights grounded in lived experience.

Patient engagement is commonly defined as the active involvement of patients and caregivers in the research process. Rather than serving solely as study participants, patients and caregivers act as partners who contribute to shaping research questions, designs, and interpretation.^4^ Engaging patients as partners in research has been increasingly recognized as a means of improving the relevance, feasibility, and accessibility of health research.^3–5^ Meaningful engagement can help ensure that research questions reflect patients’ priorities that study methods are feasible and considerate of patients’ circumstances, and that findings are interpreted and communicated in ways that are meaningful to those affected.

In response to this growing recognition, several national and international organizations have developed frameworks to promote patient-centered research. In Canada, the Canadian Institutes of Health Research’s Strategy for Patient-Oriented Research (CIHR SPOR) emphasizes inclusiveness, support, mutual respect, and co-building.^6^ In the United States, the Patient-Centered Outcomes Research Institute (PCORI) has advanced patient engagement through its Engagement Rubric, which promotes early stakeholder involvement, continuous collaboration, and co-dissemination of research findings.^7^ In the United Kingdom, the National Institute for Health Research (NIHR) has similarly emphasized public involvement through its INVOLVE program, advocating for research conducted with or by patients rather than to or for them.^8^

However, while frameworks like SPOR and PCORI articulate important values, they often provide limited guidance on how to operationalize patient engagement in specific disease contexts. This limitation is particularly evidence in esophageal cancer, where formal mechanisms for sustained patient involvement in research remain limited.^3^ Engagement efforts are frequently project-specific, short-term, and difficult to replicate or scale across institutions and healthcare systems. To address this gap, we established the Esophageal Cancer Patient Network (ECPN), a disease-specific patient engagement initiative designed as a reusable research tool rather than a single-project advisory group. The purpose of this paper is to describe the conceptual foundation, governance structure, and implementation strategy of the ECPN, positioning it as a scalable protocol for multidisciplinary and international esophageal cancer research.

## RATIONALE FOR A DISEASE-SPECIFIC ENGAGEMENT TOOL

Generic patient engagement frameworks offer valuable guiding principles but may not adequately account for the unique challenges of specific diseases. Esophageal cancer presents several characteristics that underscore the need for a tailored approach: relatively low incidence compared with other cancers, complex and invasive treatments, substantial survivorship morbidity, and marked heterogeneity in patient experiences across disease stages and healthcare systems.^1,2^

In this context, ad hoc engagement methods, such as isolated focus groups or one-time consultations, risk capturing only a narrow subset of patient perspectives. Moreover, without a standing engagement structure, patient input is often disconnected across studies, limiting continuity and the ability to build cumulative knowledge. These limitations can hinder efforts to align research priorities with patient needs and to translate findings into meaningful improvements in care.

The ECPN was conceived to address these challenges by providing sustained engagement infrastructure specific to esophageal cancer, its treatments, and survivorship. By establishing an ongoing network of patients and caregivers, the ECPN supports continuous collaboration across research projects, disciplines, and institutions. Importantly, this sustained model also enables the development of patient expertise over time. Through repeated engagement, training, and mentorship, patient partners are supported in building skills such as reviewing research materials, contributing to the formulation of research questions, and participating in discussions around study design and interpretation. This approach allows patient perspectives to inform not only individual studies but also broader research agendas within the field, representing a key advantage over project-specific engagement models which limit opportunities for longitudinal involvement.

## GOVERNANCE AND TERMS OF REFERENCE

A central component of the ECPN research tool is its formal governance structure, detailed through a Terms of Reference document that is publicly available on its website. This document defines the network’s purpose, scope, member roles and responsibilities, meeting structure, confidentiality expectations, and ethical considerations.

The Terms of Reference provide clarity for both patient partners, scientists, and clinicians, establishing shared expectations and promoting transparency. Public availability of this document facilitates recruitment, onboarding, and external collaboration, while supporting accountability and trust. Importantly, formal governance enables the ECPN to function as durable research infrastructure rather than a time-limited advisory group.

### Conceptual framework for engagement across the research lifecycle

**Fig. 1:**
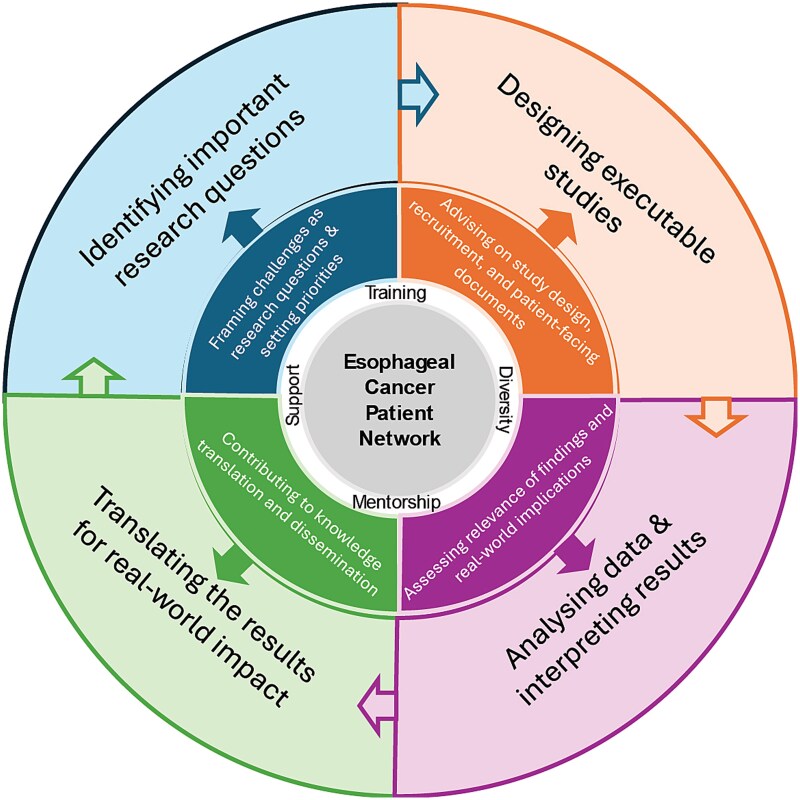
Conceptual framework for the Esophageal Cancer Patient Network (ECPN).

The ECPN is guided by a conceptual framework that situates patient engagement as a continuous process across four domains of the research lifecycle: identifying important research questions, designing executable studies, analyzing and interpreting results, and translating findings for real-world impact (see Fig. 1).

At the center of the framework is the ECPN itself, supported by four foundational pillars: training, mentorship, diversity, and support. These pillars are intended to ensure that patient partners feel comfortable working alongside scientists and clinicians in conducting research, are equipped to offer meaningful contributions, that diverse perspectives are represented, and that participation is accessible and sustainable. The framework emphasizes bidirectional collaboration and feedback loops, allowing patient insights to inform research decisions at multiple stages.

### Levels of engagement as an implementation guide

To support practical implementation, the ECPN defines five levels of patient engagement: inform, consult, involve, collaborate, and empower. Each level is characterized by a distinct patient role, scientist/clinician role, and set of example activities within the network.

These levels serve as an implementation guide rather than a rigid hierarchy, allowing research teams to tailor engagement strategies to specific study goals and contexts. By explicitly defining levels of involvement, the ECPN provides a shared language that reduces ambiguity, clarifies expectations, and supports transparent collaboration between patients and scientist/clinician.

### Implications for multidisciplinary and international research

Diseases with relatively low prevalence at a single center, such as esophageal cancer, pose unique challenges for patient engagement when efforts are limited to a single center. Individual institutions may see a small and heterogeneous patient population, making it difficult to capture the breadth of experiences necessary to inform research questions that are relevant and generalizable. As with single-center clinical studies, patient engagement confined to one institution risks reflecting local practices, referral patterns, and demographic characteristics rather than the diversity of patient experiences across healthcare systems. Developing a patient engagement model that draws on perspectives from multiple centers therefore strengthens the representativeness of patient input, enhances external validity, and supports the development of research priorities and study designs that are applicable to a wider population. A patient network that reflects multi-center experiences mirrors the rationale for multi-center research studies, allowing shared learning, comparison of care pathways, and integration of diverse lived experiences into a unified research agenda.

The ECPN was intentionally designed to be adaptable beyond a single institution or healthcare system. Its emphasis on virtual participation, flexible engagement, and transparent governance supports international collaboration and inclusion of geographically diverse patient partners. As a standing engagement platform, the ECPN can be integrated into multidisciplinary studies spanning surgery, medical oncology, radiation oncology, survivorship research, and health services research.

While developed in the context of esophageal cancer, the ECPN’s structure is designed to be transferable to other rare or complex disease areas where patient engagement remains underdeveloped. At the same time, the ECPN is intentionally structured as an open and collaborative network, welcoming participants from patients, caregivers, and scientists across institutions and regions who wish to contribute or collaborate within the existing framework.

## CONCLUSION

The ECPN represents a novel, disease-specific research tool designed to operationalize patient engagement across the esophageal cancer research lifecycle. By pairing a clear governance structure with a continuous engagement framework and defined levels of involvement, the ECPN provides practical infrastructure for integrating patient voices in a transparent, ethical, and sustained manner. The ECPN is a scalable and adaptable protocol that addresses a critical gap between high-level engagement principles and real-world implementation.

## Notes

Financial support: None.

## References

[1] Darling G E . Quality of life in patients with esophageal cancer. Thorac Surg Clin 2013; 23: 569–75. 24199706 10.1016/j.thorsurg.2013.07.011

[2] Andreassen S, Randers I, Näslund E, Stockeld D, Mattiasson A C. Patients’ experiences of living with oesophageal cancer. J Clin Nurs 2006; 15: 685–95. 16684164 10.1111/j.1365-2702.2006.01412.x

[3] Schandl A, Mälberg K, Haglund L, Arnberg L, Lagergren P. Patient and public involvement in oesophageal cancer survivorship research. Acta Oncol 2022; 61: 371–7. 34923913 10.1080/0284186X.2021.2016950

[4] Duffett L . Patient engagement: what partnering with patient in research is all about. Thromb Res 2017; 150: 113–20. 27817863 10.1016/j.thromres.2016.10.029

[5] Concannon T W, Fuster M, Saunders T et al. A systematic review of stakeholder engagement in comparative effectiveness and patient-centered outcomes research. J Gen Intern Med 2014; 29: 1692–701. 24893581 10.1007/s11606-014-2878-x PMC4242886

[6] The SPOR Evaluation Team . Evaluation of the Strategy for Patient-Oriented Research (SPOR) [Internet]. Ottawa; Canada: Canadian Institutes of Health Research. 2023; Available from: https://cihr-irsc.gc.ca/e/53635.html?utm_.

[7] Sheridan S, Schrandt S, Forsythe L, Hilliard T S, Paez K A. Advisory panel on patient engagement (2013 inaugural panel) advisory panel on patient engagement 2013 inaugural panel, Hilliard TS, Paez KA. The PCORI engagement rubric: promising practices for partnering in research. Ann Fam Med 2017; 15: 165–70. 28289118 10.1370/afm.2042 PMC5348236

[8] INVOLVE. Briefing notes for researchers - public involvement in NHS, health and social care research. Natl Inst Health Res [Internet] Version: 2.0 - May 2024; Available from: https://www.nihr.ac.uk/briefing-notes-researchers-public-involvement-nhs-health-and-social-care-research.

